# Comparison between the effects of epidural lidocaine, tramadol, and lidocaine–tramadol on postoperative pain in cats undergoing elective orchiectomy

**DOI:** 10.1186/s13028-023-00696-9

**Published:** 2023-07-11

**Authors:** Cecilia Vullo, Annastella Falcone, Gabriele Marino, Salvatore Monti, Adolfo Maria Tambella, Antonello Bufalari, Giuseppe Catone

**Affiliations:** 1 Department of ChiBioFarAm, University of Messina, Viale Ferdinando Stagno d’Alcontres, Messina, 98166 Italy; 2 Veterinary Teaching Hospital, University of Messina, Via Palatucci, Messina, 98168 Italy; 3 Department of Veterinary Sciences, University of Messina, Via Palatucci, Messina, 98168 Italy; 4 School of Biosciences and Veterinary Medicine, University of Camerino, Via Circonvallazione, Matelica, 62022 Italy; 5 Department of Veterinary Medicine, University of Perugia, Via San Costanzo, Perugia, 06126 Italy

**Keywords:** Analgesia, Cats, Epidural anesthesia, Lidocaine, Orchiectomy, Tramadol

## Abstract

**Background:**

In veterinary clinical practice, orchiectomy is one of the most common surgical procedures for cats and is performed mainly in young animals. The purpose of this study was to compare three different epidural (EP) analgesic protocols used in cats undergoing orchiectomy in order to determine which protocol resulted in superior outcomes in terms of perioperative analgesia. Twenty-one client-owned male cats were premedicated with a combination of dexmedetomidine (10 µg/kg) and midazolam (0.2 mg/kg) injected intramuscularly. Anesthesia was induced intravenously with propofol. Cats were randomly divided in three treatment groups of seven animals each: Group L received EP lidocaine (2 mg/kg), Group T received EP tramadol (1 mg/kg), and Group LT received EP lidocaine (2 mg/kg) plus tramadol (1 mg/kg). The post-operative pain level was assessed using two different scales: the Glasgow Composite Measure Pain Scale-Feline (CMPS-F) and the Feline Grimace Scale (FGS). Rescue analgesia was administered when the CMPS-F total score was ≥5 or the FGS total score was ≥4.

**Results:**

No adverse effects related to tramadol or lidocaine were observed. Based on post-operative pain assessments, significant differences between groups were observed according to both pain scoring systems. In particular, in Group LT, the CMPS-F and FGS scores decreased significantly in the first six hours following castration.

**Conclusions:**

Based on our results, EP lidocaine plus tramadol provided the best post-operative analgesic effects in cats submitted to orchiectomy lasting 6 h and could also be a choice to consider for longer surgical procedures.

## Background

Neutering is a common surgical procedure and is performed as a method of contraception to control population growth [1, 2], to reduce sexually dimorphic behaviors such as urine spraying and aggression [3, 4], and to reduce unwanted pregnancy, in addition to reducing the risk of some viral infectious diseases [5, 6].

The use of intramuscular (IM) drug combinations is the most popular choice for anesthesia in cat castration [7–13] because injectable anesthetics are preferred over inhalation-based anesthetics. Combinations of sedatives, induction agents suitable for IM administration, non-steroidal anti-inflammatory drugs, and opioids are used for this purpose [10, 14].

As an alternative to systemic analgesia, locoregional anesthesia (LA) is becoming increasingly popular in veterinary medicine, resulting in better control of perioperative pain for many surgical procedures [14]. The most common LA for orchiectomy is the intratesticular injection of local anesthetics; however, as an alternative or adjunct to improve analgesia and reduce post-operative analgesic requirements, epidural (EP) injections of local anesthetics may be used to desensitize the testicles and the spermatic cord in cats [15].

Tramadol, a synthetic analogue of codeine, is a centrally acting opioid analgesic drug [16–18]. The mechanism of action of this drug has not been fully clarified. To date, most studies have noted that tramadol has two main mechanisms of action: the activation of µ-opioid receptors and the inhibition of monoamine neurotransmitter reuptake [19–21].

In humans, the analgesic properties of tramadol are mainly correlated to the production of the drug’s active metabolite O-desmethyl tramadol (M1), which binds to µ-opioid receptors with an affinity almost 300-fold greater than that of the original compound [21]. Nevertheless, tramadol binds weakly to the µ-opioid receptors with 10-fold lower affinity than codeine and 6000-fold lower affinity than morphine [19].

Lidocaine is an amide-type local anesthetic commonly used epidurally [22–25]. Lidocaine’s mechanism of action occurs mainly through blocking sodium channels of the neural cell membrane [26]; when administered into the epidural space, this drug produces rapid desensitization with good muscle relaxation [27].

Epidural tramadol, both alone and in combination with lidocaine, has been applied in various species, and in many cases, the results have proved encouraging [28–33]. Baniadam et al. [28] considered caudal epidural tramadol sufficient to allow common surgical procedures to be performed in standing cattle. Dehkordi et al. [29] investigated the anti-nociceptive effects of epidural tramadol, tramadol–lidocaine, and lidocaine in goats and concluded that a combination of tramadol–lidocaine given via epidural injection produced an anti-nociceptive effect in the perineal region, which was rapid in onset and had a longer duration of action than lidocaine alone. In female dogs undergoing ovariohysterectomy, epidural tramadol was shown to be a safe analgesic but did not improve the analgesic effects compared to IM administration [30]. In a study performed in horses to evaluate the efficacy of epidural lidocaine combined with tramadol or neostigmine on perineal analgesia, De Rossi et al. [31] concluded that the duration of analgesia was longer with lidocaine plus tramadol compared to lidocaine plus neostigmine or lidocaine alone, and all the treatments produced a mild or moderate motor block without behavioral changes. The epidural analgesic effects of tramadol were also investigated in rabbits to determine the onset time of analgesia, the duration of flaccid paresis, and the duration of analgesia. The authors concluded that tramadol plus lidocaine prolonged epidural analgesia [32]. In the same species, it was demonstrated that the lumbosacral epidural administration of lidocaine combined with tramadol is a better choice for potentiating analgesia than the use of either drug separately and can be safely used in rabbits undergoing knee surgery [33]. In all these species, the lumbosacral epidural administration of lidocaine combined with tramadol was found to be a good alternative for enhancing postoperative analgesia.

To date, only two studies have been published on the effects of EP tramadol in cats. In the first study, the authors concluded that both morphine and tramadol provided analgesia for the first 6 h, but EP morphine resulted in longer-lasting analgesia when compared to tramadol in cats receiving standardized noxious stimulation [34]. In the second study, the results indicated that the EP administration of tramadol with lidocaine provided a longer duration of analgesia than tramadol IM in cats receiving painful mechanical stimuli through the application of pressure from hemostatic forceps [35].

On the basis of the current literature, the EP administration of a lidocaine and tramadol combination could represent a promising anesthetic technique for cats undergoing caudal abdomen surgery. The aim of this study was to compare three analgesic treatments administered epidurally at the lumbosacral level: lidocaine, tramadol, and lidocaine–tramadol. The comparison included an assessment of side effects and the perioperative analgesia in male cats undergoing castration. We hypothesized that the EP administration of lidocaine in combination with tramadol could improve post-operative analgesia in cats undergoing orchiectomy compared to the application of lidocaine or tramadol alone.

## Methods

### Animals

The study was approved by the Bioethics Committee of the Department of Veterinary Sciences of the University of Messina according to the Good Scientific Practice Guidelines and the European legislation, EU Directive 2010/63 (Ethical approval code N. 072/2021). Twenty-one healthy privately owned male cats (up to 4 years of age) undergoing elective gonadectomy and admitted to the Veterinary Teaching Hospital of the University of Messina from December 2021 to March 2022 were included in this study.

### Study design

This study was a prospective, randomized, and blinded clinical trial.

### Procedures

The preoperative health status of each cat was evaluated through a physical examination and laboratory tests. Health physical status was scored according to the American Society of Anesthesiologists classification (ASA). Cats with an ASA status above I, obese cats, and cats with a skin infection over the injection site or aggressive behavior that did not allow the preoperative clinical visit to assess the ASA score were excluded. For each cat, baseline values for heart rate (HR), respiratory rate (RR), and rectal temperature (T_r_°) were evaluated and recorded before the administration of anesthesia (baseline values).

The animals were randomly assigned to one of three groups that differed in the analgesic treatment administered epidurally: lidocaine (Group L), tramadol (Group T), and lidocaine plus tramadol (Group LT). A manual randomization technique was used to allocate cats into the three groups: sequentially numbered, opaque sealed envelopes (SNOSE approach) were used to effectively conceal the randomization sequence. Each treatment group was composed of seven cats. Food, but not water, was withheld for at least 10 h before anesthesia. All cats were premedicated with dexmedetomidine (10 µg/kg, Dexdomitor, Vetoquinol, Italy) and midazolam (0.2 mg/kg, Hameln, Italy) mixed in the same syringe and administered IM in the quadricep muscle. As soon as the cats achieved lateral recumbency and a sufficient degree of muscle relaxation (lost righting reflex), they were placed on top of an electrical heating pad (Nicrew pet heating pad) and irradiated with a heating lamp. A 20- or 22-gauge catheter (Delta Med, Italy) was then aseptically placed in a cephalic vein. Anesthesia was induced via the slow intravenous (IV) administration of propofol to minimize negative cardiac and respiratory effects (Proposure, Merial, Italy) until a loss of the palpebral reflex and mandibular tone was achieved.

The cats breathed room air.

All cats were administered lactated Ringer’s solution (3 mL/kg/hour) (Lactated Ringer’s solution, S.A.L.F., Italy) via IV during the procedure.

**Fig. 1:**
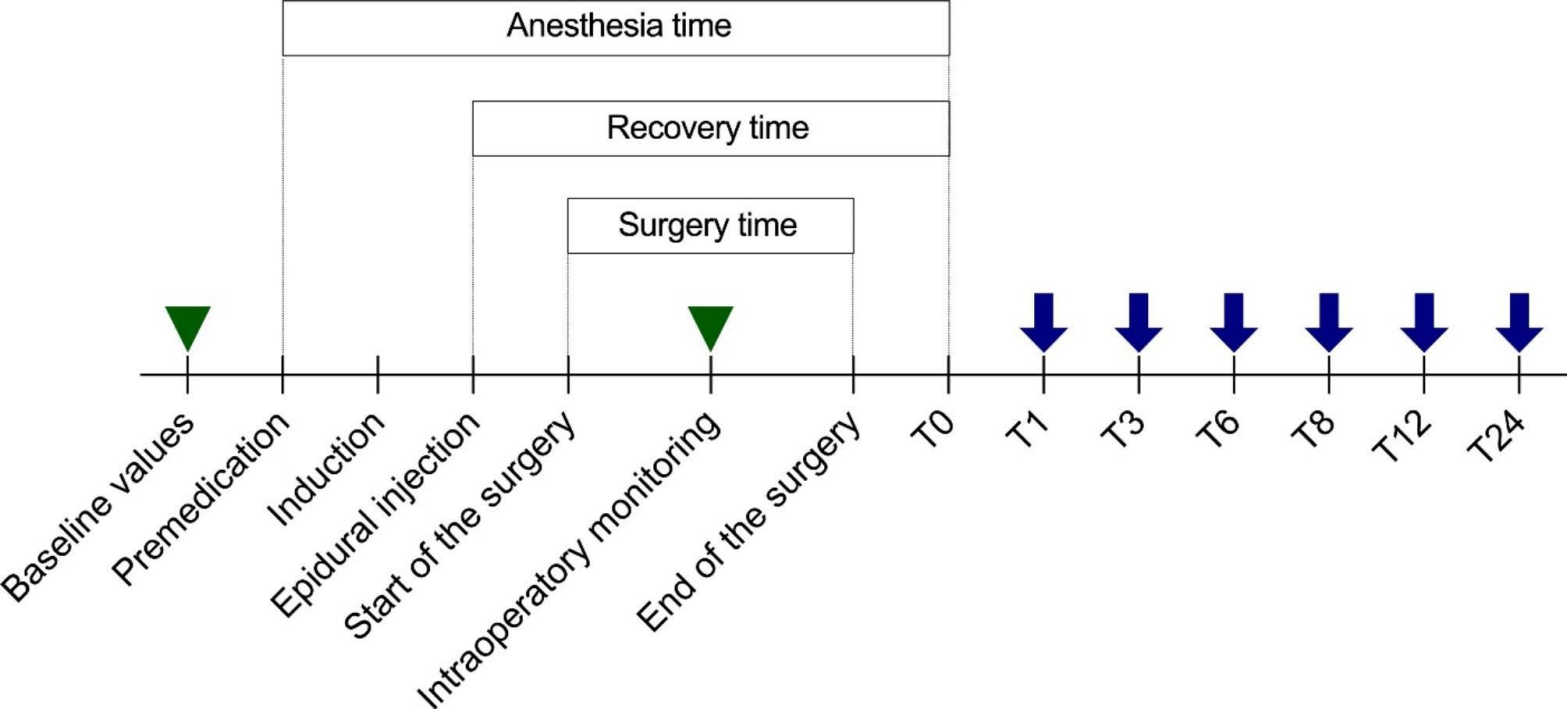
Timeline of the study with defined study points. Green triangles indicate the time points for clinical monitoring performed at baseline and during surgery (every 5 min after epidural injection). The blue arrows indicate the time points for pain assessments, performed 1 h (T1), 3 h (T3), 6 h (T6), 12 h (T12), and 24 h (T24) after the time point at which each animal regained movement and sensation in its hind limbs (T0)

The bladder was manually emptied if overly repleted, and the cats were placed in sternal recumbency, with the pelvic limbs extended cranially. The lumbosacral area was then clipped and aseptically cleaned. Next, a 22G Quincke spinal needle (Artsana, Italy) was inserted into the EP space at the lumbo-sacral junction. Epidural needle placement was confirmed by the loss-of-resistance technique with saline and by the absence of blood and/or cerebrospinal fluid in the aspirate. After confirmation of placement, animals received one of the three treatments: lidocaine 2 mg/kg (Group L) (Lidocaine 2%, ATI, Italy), tramadol 1 mg/kg (Group T) (Altadol, Formevet, Italy), or lidocaine and tramadol at the same doses (Group LT). All treatments were diluted with saline solution to a total volume of 0.22 mL/kg administered over 60 s. The staff involved in the study were three researchers. The anesthetist was unaware of the content of the syringe prepared by the two assistants. The animals were placed in sternal position with their hindlimbs adducted for 10 min after EP, and the EP block was confirmed by subsequent relaxation of the anal sphincter assessed by lightly applying pressure to the anus or perianal skin performed every 2 min for 10 min. Animals that did not experience loss of anal tone were excluded from the study, based on the assumption that EP administration had failed. As soon as the animals were connected to a multiparametric anesthetic monitor (EDAN IM50, Edan Instruments, China) recording of vital parameters was started and continued until the end of surgery. Monitoring included electrocardiogram tracing, HR, non-invasive systolic arterial blood pressure (SAP), diastolic arterial blood pressure (DAP), and mean arterial blood pressure (MAP) with an appropriate cuff size placed over the median artery of the forelimb between the elbow and carpus; RR by observing thoracic excursion; and hemoglobin oxygen saturation (SpO_2_) with the probe placed on the tongue and esophageal body temperature (T_e_°). Physiological parameters were recorded every 5 min (Fig. 1).

The cats were placed in lateral recumbency and submitted to scrotal gonadectomy using an open approach [36] undertaken by the same experienced surgeon while teaching final year veterinary students.

During the surgery, if animals had signs of inadequate anesthesia (such as an increase in the HR or RR by more than 25% compared to baseline values) or showed movements related to surgical stimulation, an additional bolus of propofol (0.5 mg/kg) and rescue analgesia with IV fentanyl (2 µg/kg, Fentadon, Dechra, Italy) were administered until reaching an adequate plane of anesthesia. If desaturation (SpO_2_ < 95%) or apnea (longer than 10 s) occurred, manually assisted ventilation was rapidly performed after orotracheal intubation. In these cases, the cats were excluded from the study. Anesthesia time (from sedation until the animals regained movement and sensation in their hind limbs), surgery time (from the scrotal midline incision to the removal of both testicles), and recovery time (the duration between epidural injection and the ability of the cats to keep their heads up and show awareness of their surroundings) were recorded. At the end of surgery, the cats were allowed to recover spontaneously without drug antagonism. The time when the cats regained sensation in their hind limbs (T0) was evaluated using a brief mild clamping stimulus on the hind limbs at 5 min intervals.

Pain assessment: Post-operative pain scores were assessed by the anesthetist, who was blinded to the treatment allocation, using two different scoring systems: the Glasgow Composite Measure Pain Scale-Feline (CMPS-F) that included 28 descriptors within 7 behavioral categories, with associated descriptive expressions, and the Feline Grimace Scale (FGS) based on changes in facial expression that consisted of five action units (ear position, orbital tightening, muzzle tension, whisker changes, and head position). Pain scores were considered as primary outcomes of the study. Pain scores were applied at 1, 3, 6, 8, 12, and 24 h (T1, T3, T6, T8, T12, and T24) after T0, i.e., the time point at which each animal showed a positive response to stimulation of the hind limbs (Fig. 1).

The cutoff score for the administration of rescue analgesia (0.3 mg/kg of IM methadone; Semfortan, Animal Health, Italy) was ≥ 5/20 for the CMPS-F and ≥ 4/10 for the FGS. Cats receiving rescue analgesia if at least one of the two scales resulted in high total score and were further monitored for signs of pain but excluded from further data analysis.

### Statistical analysis

The sample size was calculated with the G-Power software (version 3.1.9.6; Heinrich-Heine-Universität Düsseldorf, Düsseldorf, Germany) using the analysis of variance (ANOVA) method, power 90%, alpha error 0.05, and effect size (F = 1). We hypothesized that we would observe a 2-point difference between and within groups on the CMPS-F. Calculation of the total sample size for the three-group study yielded 18 patients. This number was increased to 21 patients by adding 15%, taking into account the nonparametric nature of the test to be used in the analysis of data.

Data were reported using the mean and standard deviation or standard error, median, and/or min–max range, as appropriate. The assumptions of normality in the data distribution and equality of variance were assessed using the Shapiro–Wilk and the Brown–Forsythe tests. One-way ANOVA and Holm–Sidak post-hoc tests were used to compare cardinal variables between the three groups. Longitudinal comparisons within each group were performed using a Repeated Measures ANOVA and the Holm–Sidak post-hoc test. Kruskal–Wallis’s test, Dunn’s multiple comparison test, or the Mann–Whitney test were used to compare the CMPS-F and FGS values between groups. The pain scores were also compared longitudinally within each group using a Friedman test and Dunn’s multiple comparison test. The frequency of cats requiring postoperative rescue analgesia was analyzed via Chi-square and Fisher’s exact tests. A P*-*value less than 0.05 was considered statistically significant. GraphPad Prism 9 for MacOS, version 9.3.1 (GraphPad Software Inc., San Diego, CA, USA), was used to perform the statistical analysis.

## Results

Twenty-one cats collected in four months (December 2021–March 2022) met the inclusion criteria. There was no significant difference between groups in terms of age and body weight (Table 1). The IM administration of 10 µg/kg dexmedetomidine and 0.2 mg/kg midazolam caused lateral recumbency in all cats without unconsciousness and allowed the catheter placement in the cephalic vein. The induction of anesthesia with propofol was used to achieve unconsciousness, a lack of palpebral reflex, mild jaw tone, and myorelaxation to enable proper positioning and EP needle placement by the same experienced anesthetist. No adverse effects related to treatments administered epidurally were observed, such as hypotension, hypothermia, bradycardia, or neurotoxicity. Anal tone was lost in all cats 10 min after EP administration. EP needle positioning, EP drug administration, and surgery were performed without complications in all cases. During surgery, the depth of anesthesia was monitored, as was unresponsiveness to surgery and jaw tone or myorelaxation. No significant differences were observed for the overall anesthesia time (ranging from 62 to 78 min, *p* = 0.8004), the duration of the surgery (ranging from 11 to 20 min, *p* = 0.4103), and the recovery time (ranging from 42 to 50 min, *p* = 0.8830) (Table 2).

**Table 1:** Demographic data in the three groups. Results are reported as mean ± standard deviation

	Group L	Group T	Group LT	Statistics
**Age** (months)	23.4 ± 15.6	25.4 ± 15.2	28.0 ± 14.9	*p* = 0.8554
**Weight** (kg)	4.3 ± 0.7	4.2 ± 0.6	4.8 ± 0.5	*p* = 0.2446

Legend: Group L: lidocaine group; Group T: tramadol group; Group LT: lidocaine/tramadol group

**Table 2:** Duration of the perioperative phases of the study in the three groups. Results are reported as mean ± standard deviation

	Group L	Group T	Group LT	Statistics
**Anesthesia time** (minutes)	71.7 ± 6.5	72.4 ± 2.6	70.7 ± 4.4	*p* = 0.8004
**Surgery time** (minutes)	15.1 ± 3.0	16.9 ± 2.8	15.0 ± 2.6	*p* = 0.4103
**Recovery time** (minutes)	45.0 ± 3.3	44.4 ± 1.6	44.4 ± 2.2	*p* = 0.8830

Legend: Group L: lidocaine group; Group T: tramadol group; Group LT: lidocaine/tramadol group

During surgery, eight animals (three in group L, two in group T, and three in group LT) showed a slight return of the palpebral reflex with the eyeballs, which modified the central position from the ventromedial position, but no animals required any intra-operative rescue analgesia or further anesthetic drugs to deepen the anesthetic plan. It was also not necessary to intubate any animal because the RR was never lower than 25% compared to baseline values, and SpO2 was never less than 95%, except for one cat in Group L that presented 94% SpO_2_ in which a new better reading was attempted after repositioning the pulse oximeter probe.

During anesthesia, cats in all treatment groups were placed over the same heating pad and presented similar T_e_°(Table 3), except for the temperature five minutes after EP (*p* = 0.0151), when multiple comparison analysis showed a higher temperature in Group T than that in Group L (*p* = 0.0148). A gradual tendency for T_e_° to decrease was observed during anesthesia in all groups (Group L: *p* < 0.0001; Group T: *p* < 0.0001; Group LT: *p* < 0.0001).

**Table 3:** Physiological parameters at baseline and during surgery in the three groups

		Group L	Group T	Group LT	*Statistics between groups*	*Multiple comparison analysis*
**T°** (°C)	BV	38.2 ± 0.4 38.2 (37.8–39.0)	38.7 ± 0.1 38.7 (38.5–38.8)	38.5 ± 0.4 38.50 (37.8–39.0)	*p = 0.0693*	
	5 min	37.8 ± 0.5 37.9 (37.2–38.8)	38.5 ± 0.2 38.6 (38.3–38.7)	38.3 ± 0.5 38.4 (37.6–38.8)	*p = 0.0151*	*LvsT: p < 0.05*
	10 min	37.7 ± 0.5 37.8 (37.2–38.7)	38.3 ± 0.2 38.3 (38.0-38.6)	38.2 ± 0.5 38.3 (37.40–38.70)	*p = 0.0596*	
	15 min	37.6 ± 0.5 37.6 (37.0-38.6)	38.1 ± 0.2 38.0 (37.8–38.4)	38.1 ± 0.4 38.2 (37.4–38.5)	*p = 0.0537*	
	20 min	37.5 ± 0.5 37.4 (37.0-38.5)	37.9 ± 0.1 38.0(37.7–38.1)	37.9 ± 0.4 38.0 (37.2–38.4)	*p = 0.0697*	
	***Statistics within each group***	*p < 0.0001*	*p < 0.0001*	*p < 0.0001*		
** h** (Beats per minute)	BV	175.4 ± 6.0 175 (168–185)	163.4 ± 8.4 162 (150–174)	146.0 ± 12.6 146 (130–170)	*p < 0.0001*	*LvsT: p < 0.05* *LvsLT: p < 0.0001* *TvsLT: p < 0.01*
	5 min	132.9 ± 4.4 132 (128–140)	140.0 ± 17.0 130 (120–162)	132.3 ± 13.3 130 (118–158)	*p = 0.4642*	
	10 min	129.0 ± 4.6 128 (124–136)	127.6 ± 19.6 120 (110–155)	120.6 ± 10.7 120 (110–142)	*p = 0.4559*	
	15 min	128.1 ± 4.8 128 (122–135)	118.6 ± 14.6 120 (100–140)	119.1 ± 10.3 120 (110–138)	*p = 0.0535*	
	20 min	127.0 ± 4.4 128 (120–132)	118.6 ± 14.6 110 (100–140)	114.9 ± 10.7 116 (100–134)	*p = 0.1251*	
	***Statistics within each group***	*p < 0.0001*	*p < 0.0001*	*p < 0.0001*		
**RR** (Breaths per minute)	BV	62.0 ± 6.8 62 (50–70)	50.1 ± 9.9 48 (40–66)	61.4 ± 8.1 66 (50–70)	*p = 0.0262*	*LvsT: p < 0.05* *TvsLT: p < 0.05*
	5 min	31.4 ± 2.9 32 (28–35)	43.1 ± 9.4 40 (30–60)	52.0 ± 6.7 50 (44–60)	*p = 0.0001*	*LvsT:p < 0.05* *LvsLT: p < 0.0001* *TvsLT: p < 0.05*
	10 min	28.7 ± 2.5 28 (26–33)	38.9 ± 9.4 34 (30–58)	49.1 ± 5.5 48 (42–58)	*p < 0.0001*	*LvsT: p < 0.05* *LvsLT: p < 0.0001* *TvsLT: p < 0.05*
	15 min	26.6 ± 2.5 28 (24–30)	36.0 ± 6.7 34 (28–50)	45.9 ± 6.5 48 (38–55)	*p < 0.0001*	*LvsT: p < 0.01* *LvsLT: p < 0.0001* *TvsLT: p < 0.01*
	20 min	26.0 ± 2.8 26 (22–30)	32.3 ± 3.9 32 (28–40)	43.1 ± 4.7 44 (38–50)	*p < 0.0001*	*LvsT: p < 0.01* *LvsLT: p < 0.0001* *TvsLT: p < 0.001*
	***Statistics within each group***	*p < 0.0001*	*p < 0.0001*	*p < 0.0001*		
**SpO2** (%)	5 min	98.0 ± 1.3 98 (96–100)	97.0 ± 1.6 96 (95–99)	97.1 ± 1.7 97 (95–100)	*p = 0.4398*	
	10 min	97.4 ± 1.9 98 (94–100)	97.6 ± 1.4 98 (96–100)	97.9 ± 1.5 98 (96–100)	*p = 0.8792*	
	15 min	98.1 ± 0.9 98 (97–99)	97.3 ± 1.8 98 (95–100)	98.0 ± 1.1 98 (96–99)	*p = 0.4550*	
	20 min	98.0 ± 0.8 98 (97–99)	97.7 ± 1.1 98 (96–99)	97.6 ± 1.3 98 (96–99)	*p = 0.7562*	
	***Statistics within each group***	*p = 0.5433*	*p = 0.6557*	*p = 0.6586*		
**SAP** (mmHg)	5 min	142.0 ± 4.4 140 (138–150)	142.4 ± 3.6 143 (138–148)	142.6 ± 4.6 140 (138–150)	*p = 0.9656*	
	10 min	141.9 ± 7.0 142 (132–154)	141.3 ± 4.7 141 (135–150)	140.0 ± 3.0 140 (136–146)	*p = 0.7911*	
	15 min	142.1 ± 8.3 142 (128–156)	140.3 ± 6.7 140 (132–154)	138.1 ± 2.5 138 (134–141)	*p = 0.5077*	
	20 min	142.0 ± 6.9 142 (130–152)	141.9 ± 7.4 140 (132–152)	138.0 ± 5.9 136 (128–146)	*p = 0.4688*	
	25 min	141.1 ± 5.6 140 (135–148)	140.3 ± 7.4 140 (130–150)	136.3 ± 3.5 135 (134–144)	*p = 0.2661*	
	***Statistics within each group***	*p = 0.9599*	*p = 0.8320*	*p = 0.0841*		
**MAP** (mmHg)	5 min	91.6 ± 3.8 91 (87–98)	91.0 ± 3.0 91 (86–96)	89.9 ± 2.6 90 (86–94)	*p = 0.5966*	
	10 min	90.9 ± 1.9 90 (88–94)	89.9 ± 2.3 89 (88–94)	88.4 ± 2.4 88 (85–92)	*p = 0.1573*	
	15 min	89.1 ± 1.9 90 (86–92)	89.4 ± 2.5 90 (86–94)	88.4 ± 3.1 89 (84–93)	*p = 0.7571*	
	20 min	88.9 ± 1.9 90 (86–91)	88.4 ± 1.4 88 (86–90)	88.1 ± 1.5 88 (86–90)	*p = 0.7036*	
	25 min	88.6 ± 2.1 88 (86–92)	88.6 ± 2.1 88 (85–92)	88.0 ± 2.1 88 (85–90)	*p = 0.8463*	
	***Statistics within each group***	*p = 0.0924*	*p = 0.0685*	*p = 0.3652*		
**DAP** (mmHg)	5 min	71.0 ± 5.0 70 (65–78)	69.3 ± 1.5 69 (68–72)	70.6 ± 4.0 70 (66–78)	*p = 0.6866*	
	10 min	71.4 ± 8.5 70 (60–85)	69.9 ± 2.8 70 (65–74)	70.4 ± 3.2 71 (65–75)	*p = 0.8643*	
	15 min	71.3 ± 5.5 68 (66–78)	70.1 ± 2.5 70 (66–73)	69.1 ± 2.3 70 (66–73)	*p = 0.5717*	
	20 min	70.6 ± 5.3 68 (64–78)	69.4 ± 2.1 70 (65–72)	69.6 ± 2.6 68 (68–75)	*p = 0.0637*	
	25 min	70.4 ± 3.9 70 (65–77)	68.7 ± 2.3 70 (66–72)	69.4 ± 2.1 70 (66–73)	*p = 0.6012*	
	***Statistics within each group***	*p = 0.9214*	*p = 0.5364*	*p = 0.6285*		

T°: body temperature; HR: heart rate; RR: respiratory rate; SpO_2_: arterial oxygen saturation SAP; systolic arterial blood pressure; MAP: mean arterial blood pressure; DAP: diastolic arterial blood pressure; Group L: lidocaine group; Group T: tramadol group; Group LT: lidocaine/tramadol group. BV: baseline values; 5, 10, 15, 20, and 25 min: intraoperative time point assessments measured in minutes after epidural injection. Data are shown as the mean ± standard deviation on the first line and as median (range) on the second line

Heart rate values were significantly different between groups at baseline, but these differences were clinically not relevant. During anesthesia, the HR of the three groups presented non-statistically different values (Table 3). A downward trend was also found in the HR during anesthesia (Group L: *p* < 0.0001; Group T: *p* < 0.0001; Group LT: *p* < 0.0001).

Throughout the anesthetic procedure, Group LT had higher RR values than the other groups (Group LT vs. Group T: *p* < 0.05; Group LT vs. Group L: *p* < 0.0001), while Group L had lower RR values than the other groups (Group L vs. Group T: *p* < 0.05; Group L vs. Group LT: *p* < 0.05) (Table 3). A gradual tendency for RR to decrease under anesthesia was also observed in all groups (Group L: *p* < 0.0001; Group T: *p* < 0.0001; Group LT: *p* < 0.0001). No differences were observed, between groups or within each group, in arterial blood pressure (Table 3).

All cats recovered motor activity and sensation in their hind limbs (T0) between approximately 15 and 18 min after surgery. None of the cats required emergency reversal of any drugs.

**Fig. 2:**
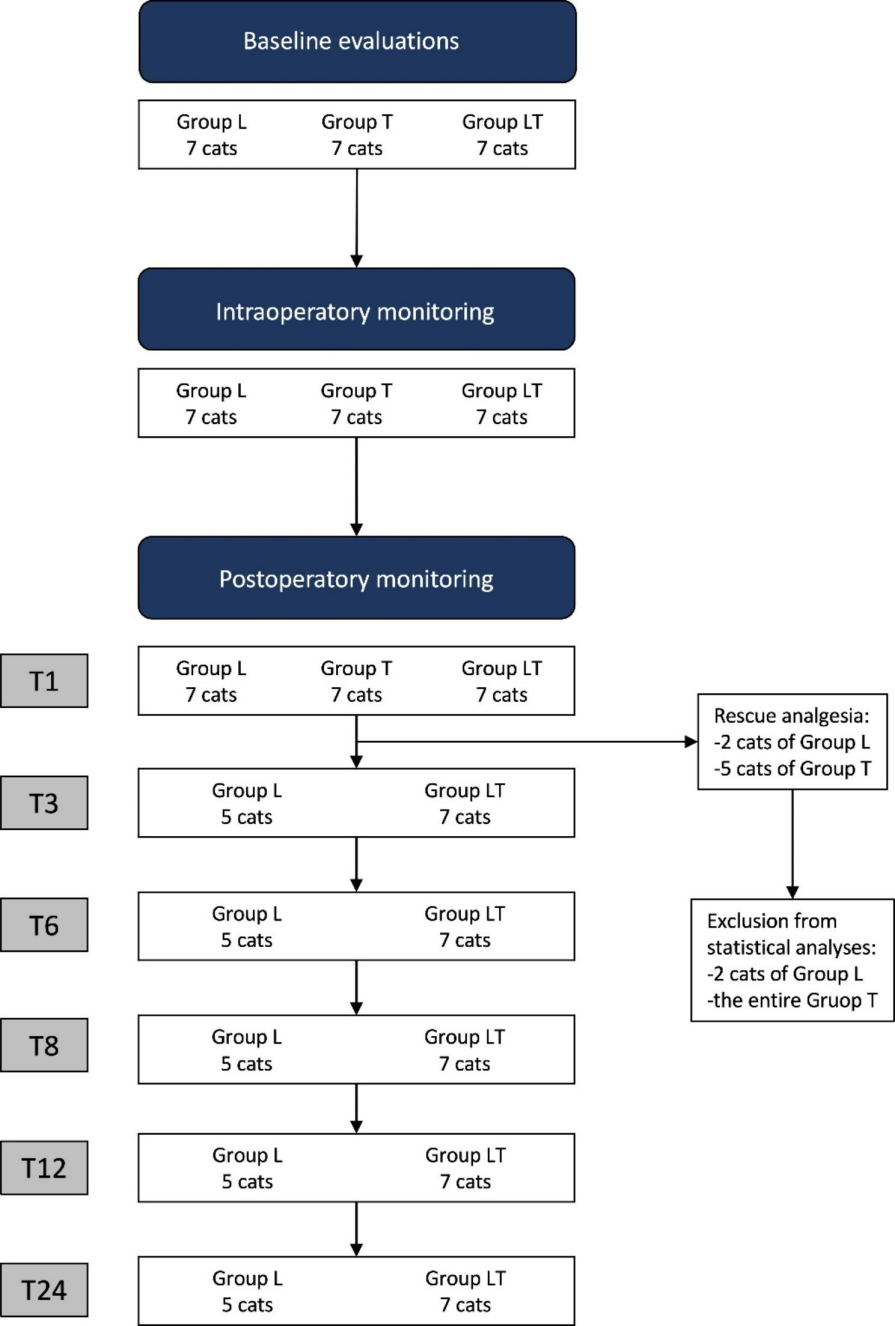
Flowchart showing the population considered for statistical analysis at each phase of the study

Post-operative rescue analgesia was administered at T1 to five, two, and no cats in Groups T, L, and LT, respectively. This difference was significant between Group T and Group LT (*p* = 0.0210). The two cats in Group L requiring rescue analgesia and the entirety of Group T were excluded from further statistical analyses (nine cats in total) but they were nevertheless monitored to assess any possible altered behavior indicative of pain, and they no longer required additional rescue analgesia (Fig. 2).

**Fig. 3:**
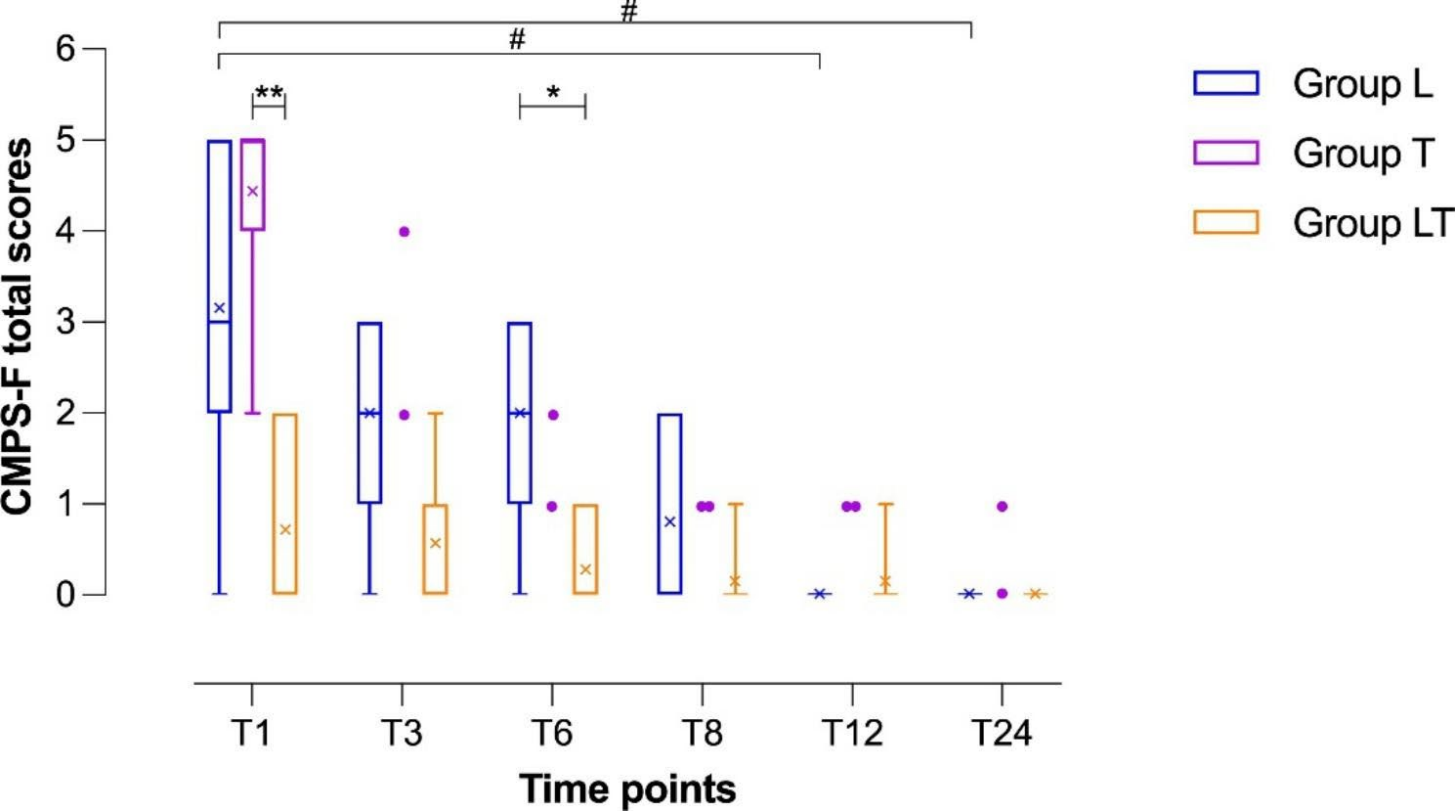
Boxplot showing the postoperative pain scores using the Glasgow Composite feline acute Pain Scale (CMPS-F) in the three groups. Group L: lidocaine group; Group T: tramadol group; Group LT: lidocaine/tramadol group. Time points indicate the time, measured in hours, after the time point at which each animal regained movement and sensation in its hind limbs, T1: 1 h; T3: 3 h; T6: 6 h; T12: 12 h; T24: 24 h. The ends of the whiskers show minimum and maximum score values; boxes show the median, first, and third quartiles; blue, violet, and orange “ｘ” signs show the mean values. Violet-colored dots indicate individual scores recorded after T1 from Group T cats that did not require rescue analgesia; the entire Group T was excluded from further statistical analyses after T1, but the two cats not requiring rescue analgesia continued being monitored for pain. Asterisks indicate statistical significance between groups. Hashtags indicate statistical significance within each group. P-values, */#: P < 0.05; **: P < 0.01

**Fig. 4:**
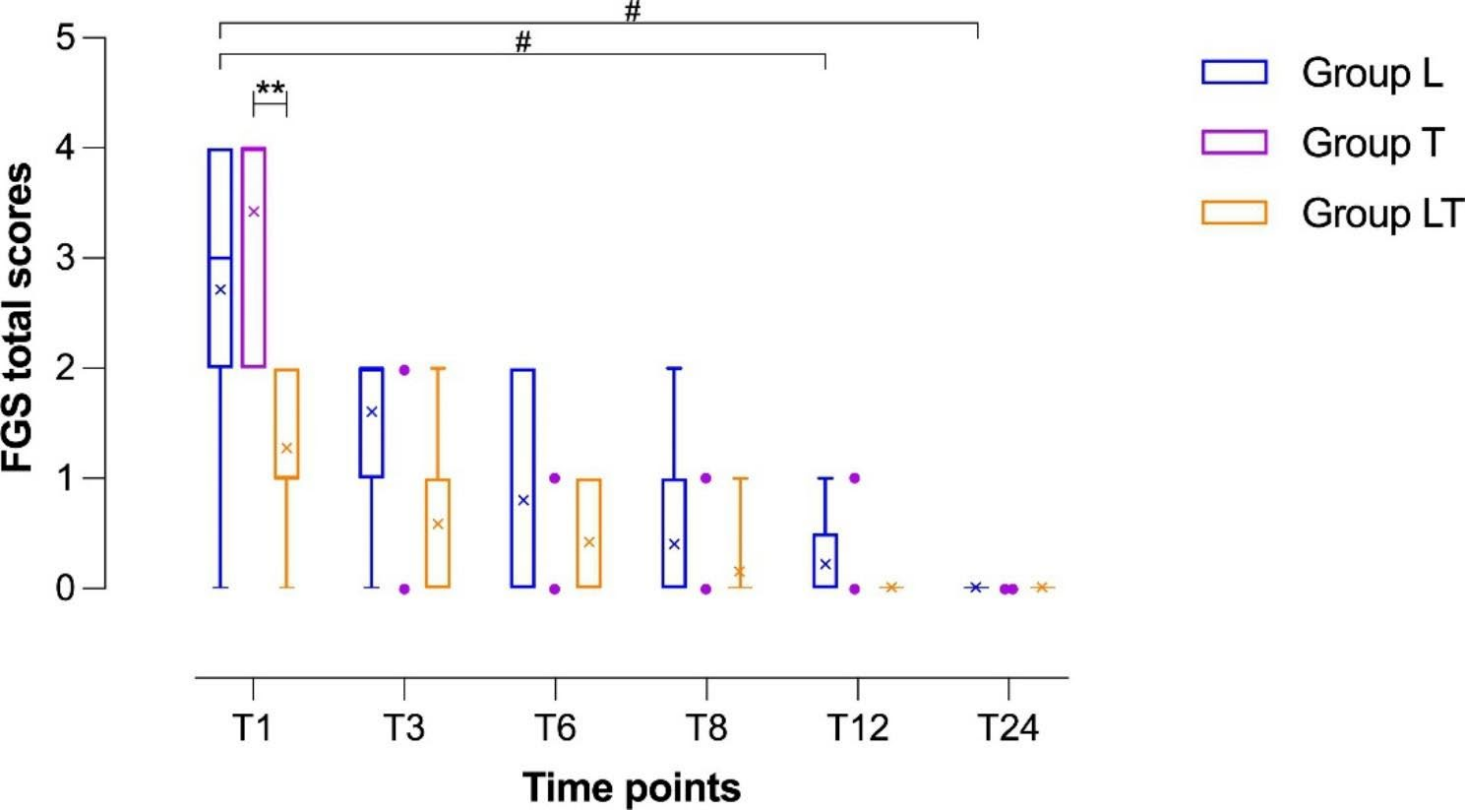
Boxplot showing the postoperative pain scores using the Feline Grimace Scale (FGS) in the three groups. Group L: lidocaine group; Group T: tramadol group; Group LT: lidocaine/tramadol group. Time points indicate the time, measured in hours, after the time point at which each animal regained movement and sensation in its hind limbs, T1: 1 h; T3: 3 h; T6: 6 h; T12: 12 h; T24: 24 h. The ends of the whiskers show minimum and maximum score values; boxes show the median, first, and third quartiles; blue, violet, and orange “ｘ” signs show the mean values. Violet-colored dots indicate individual scores recorded after T1 from Group T cats that did not require rescue analgesia; the entire Group T was excluded from further statistical analyses after T1, but the two cats not requiring rescue analgesia continued being monitored for pain. Asterisks indicate statistical significance between groups. Hashtags indicate statistical significance within each group. P-values, #: P < 0.05; **: P < 0.01

Based on post-operative pain assessment, significant differences between groups were observed according to both pain scoring systems. In particular, Group LT presented a lower pain score than the other groups. This difference between groups was found to be significant at T1 using both scoring systems (CMPS-F: p = 0.0007; FGS: *p* = 0.0048). At T1, for intergroup multiple comparisons, the cats in Group LT presented lower pain scores than those in Group T (CMPS-F, *p* = 0.0019; FGS, *p* = 0.0074), while those in Group L showed no significant differences compared to the scores in both other groups (Figs. 3 and 4).

At T3, Group LT showed a non-significantly lower pain score than Group L (CMPS-F, *p* = 0.0694).

At T6, the pain score was significantly lower in Group LT than in Group L (CMPS-F, *p* = 0.0227).

Starting from T8 and throughout the 24-hour follow-up, no significant difference between groups was observed (Figs. 3 and 4).

All groups showed a post-operative trend towards a progressive reduction of pain scores. Group L (*p* = 0.0029) showed an overall significant reduction in CMPS-F, especially from T1 to T12 (*p* = 0.0112) and from T1 to T24 (*p* = 0.0112).

A similar trend was shown using the FGS scale, with an overall significant decrease in Group L (*p* = 0.0079), especially from T1 to T12 (*p* = 0.0225) and from T1 to T24 (*p* = 0.0142). The reduction in the trend of pain scores observed in the LT group during follow up was not significant under both scoring systems; however, as noted above, the LT group already presented a lower score than the other groups at T1.

**Fig. 5:**
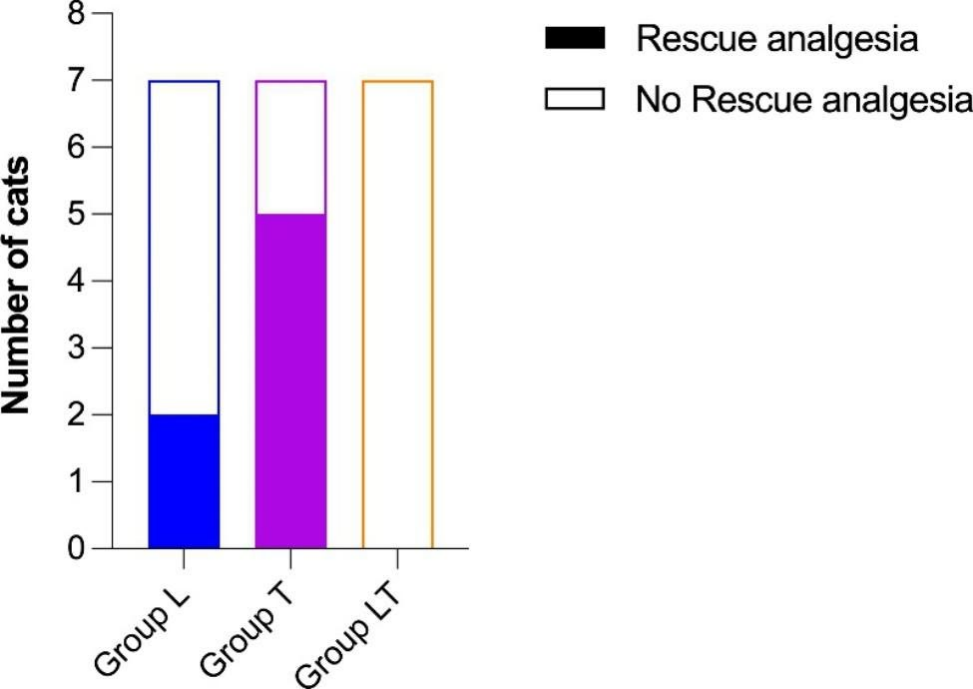
Frequency of cats requiring postoperative rescue analgesia in the three groups. Color filled bars indicate the number of cats requiring rescue analgesia; bars not filled with color indicate the number of cats that have not needed it

An overall difference was found between the groups in terms of the need for rescue analgesia after surgery (*p* = 0.0171) (Fig. 5).

## Discussion

Pain management has become an essential part of feline practice with fundamental benefit to feline health and welfare [37]. Multimodal analgesia that includes both systemically and locally/regionally administered drugs is generally the most effective method to provide pain relief, and the lumbo-sacral EP deposition of anesthetic and analgesic drugs is commonly performed in small animals as an effective means of improving anesthesia and/or analgesia [38].

The primary aim of this study was to verify whether the administration of EP lidocaine and tramadol (Group LT) resulted in better post-operative analgesia in terms of analgesia and behavioral changes indicative of pain compared to the administration of lidocaine (Group L) or tramadol (Group T) in cats undergoing orchiectomy.

Heart rate values presented statistically significant, but not clinically relevant, differences between groups at baseline. probably related to the different temperament and in any case within the physiological ranges for awake cats.

A gradual tendency to decrease during anesthesia was also observed for RR in all groups, but this downward trend of RR could be considered irrelevant from a clinical point of view, while oxygen administration, even if only by face mask, remains an appropriate preventive measure to limit hypoxemia when propofol is used for short-term procedures. No differences between groups or study time points within each group were found for SpO_2_.

With regard to the haemodynamic responses between groups, although a previous study reported a significant decrease in DAP and MAP in cats following EP tramadol [35], in the present study, no animals showed hypotension. These results are in line with those obtained by other authors who reported that arterial pressure did not present considerable changes after EP tramadol or lidocaine administration [39–41].

Before and during surgery, the animals were placed over the same heating pad. However, a gradual tendency for T_e_°to decrease under anesthesia was observed in all groups. This result could be related not only to the tendency of cats to rapidly lose temperature under anesthesia but also to a secondary effect of the epidural, which, by blocking the sympathetic nerves and producing vasodilation, accelerates this event [42]. During surgery, the depth of anesthesia was monitored by checking unresponsiveness to surgery and assessing jaw tone and myorelaxation, although myorelaxation can occur simply due to effective motor blocking linked to the epidural, as demonstrated by the fact that eight animals (three in group L, two in group T, and three in group TL) showed a slight palpebral reflex return. An additional bolus of fentanyl or propofol was not administered in these animals because they were unresponsive to surgery.

The dose of tramadol administered epidurally alone or in combination with lidocaine was 1 mg/kg, as reported by Castro et al. [33], who compared the analgesic effects of epidural tramadol with those of morphine in six healthy cats. The dose of lidocaine administered epidurally was 2 mg/kg, as reported by Lawal and Adetunji [41].

Since the use of local anesthetics is limited by their duration of action, adjuvants such as tramadol, opioids, vasoconstrictors, or α_2_ adrenoceptor agonists are often associated with local anesthetics due to their synergistic effect of extending the duration of sensory-motor blocking and increasing the quality of analgesia [43–46]. In a previous study, the addition of tramadol to epidural lidocaine prolonged the analgesic effect from 53 to 120 min in cats [35]. Similarly, in the LT Group, the addition of tramadol prolonged the efficacy and analgesia compared to the L and T Groups, without significant differences in recovery time between groups. Thus, our results indicate that lidocaine plus tramadol administered epidurally provided longer and more profound analgesic effect during the first 6 h compared to tramadol or lidocaine alone.

In clinical practice, epidural anesthesia is performed under general anesthesia, especially in cats because of their tendency to resist handling during even the simplest clinical procedure. Moreover, the benefit for performing an epidural is to limit the use of inhalants during maintenance and their undesirable effects, such as decreased cardiac output, systemic vascular resistance, or both, resulting in hypotension [13, 47–49].

Therefore, although in this study only ASA I cats were enrolled, the epidural technique may be performed safely in cats with cardiovascular problems and renal or hepatic dysfunction that could necessitate the use of light anesthesia and avoiding the use of inhalants.

Although the EP technique was performed in this study by an experienced anesthetist, this method is easy to learn, simple, and has a low incidence of complication [50, 51]. Additionally, this technique can be performed by private practitioners following a short learning period as an alternative to intratesticular blocking, which is an easy tool but enables only short-term analgesia. FGS, which was recently introduced in the feline species [52], was found to be an easy tool to use and not time-consuming for pain evaluation in cats undergoing castration. This tool is similar to the CMPS-F, which has been used for the longest time in veterinary medicine but, in the opinion of the authors, requires more time to be compiled. However, this study was not intended to compare the two pain scales.

There are some important limitations to recognize in this study. Firstly, the use of dexmedetomidine in the premedication protocol could be considered a component of analgesic management, due to the fact that both sedation and analgesia can last 40–90 min [53], and there was a short period between the IM administration of dexmedetomidine and the start of pain evaluation. However, the dose administered in the study was low, and therefore may have had a shorter period of action. Furthermore, the IM dexmedetomidine-midazolam combination is recommended in combination with an opioid [54], which was deliberately not included in this study with the aim of evaluating the analgesic effect of the EP. In addition, in this study, a control group with EP saline was not included. A control group would have likely accounted for the need of propofol and fentanyl during surgery.

Moreover, a greater number of enrolled animals would have been necessary, given the fact that in this study, animals that required rescue analgesia were excluded in the post-operative period. The calculation of sample size considered, a priori, a 15% increase due to the non-parametric nature of the tests to be applied in the assessment of postoperative pain scores, but it did not consider potential losses of cats for subsequent evaluations due to the administration of rescue analgesia, which later occurred during the study in groups T and L. For seven cats (two in Group L, five in Group T, and none in Group LT) administered methadone, there were ethical reasons to limit the pain of the cats. This factor could be considered a limitation of this study, as it decreased the number of patients evaluated for pain scores after T1. The decision to exclude these cats from the analysis of pain score data was made because all subsequent pain scores in Group T and Group L would have been underestimated through the administration of methadone. Additionally, the exclusion of the two animals with the worst pain in group L likely altered the differences between L and LT after this time point. In any case, the greater analgesic effect of the LT group compared to the other two groups remained evident.

The choice of methadone for rescue analgesia was based on the most commonly used drugs at the Veterinary Teaching Hospital where the study was performed. Although non-steroidal anti-inflammatory drugs (NSAIDs) are effective analgesics in the perioperative period, they were not considered alternatives to methadone in this study because cats are especially sensitive to the side effects of NSAIDs [54–56].

## Conclusions

The results of the present study suggest that all three treatments offered satisfactory antinociceptive effects during surgery without adverse effects such as hypotension, hypothermia, bradycardia, or neurotoxicity. However, analgesia produced via an EP lidocaine/tramadol combination provided a better analgesic effect than that of EP lidocaine or tramadol alone; this superior effect lasted for the first six hours after surgery. Ultimately, a combination of lidocaine plus tramadol improved the quality of recovery in cats that underwent orchiectomy, without the need to administer additional analgesic drugs. Therefore, the EP administration of lidocaine/tramadol could be a better choice in cats for surgical procedures longer than an orchiectomy, such as orthopedic surgery (in the pelvis and pelvic limbs) and soft tissue surgery (perianal and perineal regions, cesarean section, and urethrostomy).

## Data Availability

The datasets used and/or analyzed during the present study are available from the corresponding author upon reasonable request.

## Abbreviations

ANOVA: Analysis of variance
ASA: American Society of Anesthesiologists
CMPS-F: Composite Measure Pain Scale-Feline
DAP: Diastolic arterial pressure
ECG: Electrocardiogram
EP: Epidural
F: Result of ANOVA test
FGS: Feline Grimace Scale
H: Result of Kruskal–Wallis’s test
HR: Heart rate
IM: Intramuscularly
IV: Intravenously
L: Lidocaine
LT: Lidocaine plus tramadol
M1: O-desmethyl tramadol
MAP: Mean arterial pressure
RR: Respiratory rate
SAP: Systolic arterial pressure
SpO_2_: Arterial oxygen saturation
T: Tramadol
T_e_°: Esophageal body temperature
T_r_°: Rectal temperature
U: Result of Mann–Whitney test
χ^2^: Result of the Chi-square test
χ^2^_r_: Result of the Friedman test
